# Taurine Prevents Ibuprofen-Induced Gastric Mucosal Lesions and Influences Endogenous Antioxidant Status of Stomach in Rats

**DOI:** 10.1100/tsw.2004.207

**Published:** 2004-12-06

**Authors:** T. Balasubramanian, M. Somasundaram, A. John William Felix

**Affiliations:** ^1^ Department of Physiology, Rajah Muthiah Medical College, Annamalai University, Annamalainagar-608 002, Tamilnadu, India; ^2^ Department of Community Medicine, Rajah Muthiah Medical College, Annamalai University, Annamalainagar-608 002, Tamilnadu, India

**Keywords:** antioxidant, catalase, free radical, gastric mucosal lesion, gastric ulcer, glutathione, glutathione peroxidase, ibuprofen, lipid peroxidation, NSAIDs, reactive oxygen species, superoxide dismutase, taurine, TBARS

## Abstract

Recently, free radical—induced tissue damage is implicated in the nonsteroidal anti-inflammatory drugs (NSAIDs)—involved gastric mucosal lesion. Administration of taurine, an endogenous antioxidant, is reported to be beneficial in various clinical conditions. Therefore, we decided to study the protective effect of taurine in ibuprofen-induced gastropathy and the effects of administration of taurine on the endogenous antioxidant enzymes such as superoxide dismutase (SOD), catalase (CAT), and glutathione peroxidase (GPX), and reduced glutathione (GSH) of stomach. In rats, administration of taurine orally for three consecutive days (250 mg/kg body weight) protected the gastric mucosa from ibuprofen-induced, acute gastric mucosal lesion. In ibuprofen-treated rats, the lipid peroxidation measured as thiobarbituric acid reactive substances (TBARS), a marker for free radical—induced tissue damage, is also significantly decreased by taurine. Ibuprofen treatment resulted in a significant increase in the activities of total SOD, manganese SOD (Mn-SOD), and GPX and reduced GSH. Taurine administration in ibuprofen-treated rats also showed a significant increase in the activities of the antioxidant enzymes namely total SOD, Mn-SOD, GPX, CAT, and the level of reduced GSH. The activity of copper-zinc SOD enzyme (Cu-Zn SOD) is not affected by ibuprofen or taurine. There is no temporal relation between the antioxidant status of the stomach and the tissue damage following oral administration of ibuprofen or taurine.

### INTRODUCTION

Nonsteroidal anti-inflammatory drugs (NSAIDs) are widely used clinically as anti-inflammatory analgesic agents, but gastrointestinal injury is one of the most serious adverse effects attributable to NSAIDs[1]. Several treatment options are available to minimize gastrointestinal toxicity from NSAIDs: antacids, mucosal coating agents, prostaglandin analogs, $H_2$-receptor blockers, proton pump inhibitors, and cyclooxygenase-2 (COX-2)–selective NSAIDs. The merits and demerits of these treatment options are discussed in a number of reviews[2,3,4]. These treatments aim mostly to treat and not to prevent NSAIDs gastropathy. The recent concept that NSAIDs gastropathy is the result of neutrophil activation and subsequent release of reactive oxygen species (ROS) such as superoxide anions ($O_2^-$), hydroxyradicals ($OH^-$), and hydrogen peroxide ($H_2O_2$) throws the doors open for a new area of research in the prevention of NSAIDs gastropathy[5,6,7,8]. ROS are normally neutralized by efficient antioxidant systems in the body that include the antioxidant enzymes like superoxide dismutase (SOD), catalase (CAT), and glutathione peroxidase (GPX), and the nutrient-derived, small antioxidant molecules like vitamin E, vitamin C, carotenes, flavonoids, reduced glutathione (GSH), uric acid, and taurine[9,10]. Many studies on the use of the antioxidant enzymes such as SOD, CAT, and GPX or the nutrient-derived antioxidants like vitamin E, vitamin C, flavonoids, or GSH to minimize gastrointestinal toxicity from NSAIDs are reported in the literature[6,11,12,13,14,15,16,17,18,19,20]. Taurine (2-aminoethane sulfonic acid), an endogenous antioxidant present in high concentration in extracellular medium, is the major antioxidant that helps to prevent the accumulation of oxidants following neutrophil activation[21]. Thus, taurine supplementation could be the preferred antioxidant to prevent ROS-induced tissue injury following neutrophil activation. A number of studies about the antioxidant effect of taurine in the lung[22], heart[23], liver[24], and kidney[25] have been reported in the literature, but the beneficial effects of taurine in NSAIDs-induced gastropathy have not been well documented. The present study was undertaken to explore the protective role of orally administered taurine in NSAIDs-induced gastropathy in rats. Experimental studies on NSAIDs gastropathy have widely used either indomethacin or aspirin. Production of experimental gastric ulcer using ibuprofen is very rare and we have used ibuprofen to induce gastric lesions. The macroscopic gastric mucosal lesion is estimated as ulcer index[26] and the level of lipid peroxidation, a marker for free radical–induced tissue injury, is expressed as thiobarbituric acid reactive substances (TBARS)[27]. We have also tried to assess the possible influence of taurine on other endogenous antioxidants namely SOD, GPX, CAT, and GSH of the stomach.

### MATERIALS AND METHODS

Wistar rats of either sex weighing 200–250 g were used. The animals were maintained on standard chow diet and allowed tap water *ad libitum*. The Indian National Science Academy's guidelines for the care and use of laboratory animals were followed. Ibuprofen was used in the dose of 200 mg/kg body weight to induce acute gastric mucosal lesion as reported by Scheiman et al. in their study on the gastroprotective effect of somatostatin analog octreotide[26]. The same dose of ibuprofen was used in our study. Taurine was administered orally for three consecutive days in a dose of 250 mg/kg body weight as reported by Son et al. in their study using indomethacin[14]. The animals were divided into four groups of ten each. Group 1 served as control and received normal saline through intragastric intubation for three consecutive days. Group 2 served as taurine treated and received taurine (250 mg/kg body weight as 2.5% solution in normal saline) through intragastric intubation for three consecutive days. Group 3 served as ibuprofen treated. It was similar to Group 1, but it was treated with ibuprofen also. Group 4 served as taurine cum ibuprofen treated. It was similar to Group 2, but it was treated with ibuprofen also.

The animals were fasted for 24 h before sacrificing. During fasting, tap water was allowed *ad libitum*. Throughout the experimental period, animals were housed singly in cages with raised platforms with wide wire mesh to ensure immediate passage of all feces from the cage, to prevent coprophagy and to prevent fighting and mutilation.

Gastric mucosal lesions were induced in Group 3 and Group 4 animals by intragastric administration of 200 mg/kg body weight of ibuprofen suspended in 1% carboxymethyl cellulose in distilled water. The ibuprofen was administered 6 h before sacrificing. The animals were sacrificed using an overdose of ether. Under the conditions used, the stomachs of all animals were empty of food remnants. The abdomen was opened using a midline incision. The stomach was dissected out and opened along its greater curvature, washed with cold distilled water, and spread on cardboard with mucosal surface upwards avoiding corrugation. A transparent sheet showing millimeter scale was placed over the stomach and the total area of the gastric mucosa and the area of lesions were measured manually. The area of gastric mucosal lesions was quantitatively expressed as ulcer index (UI) (percentage lesioned area relative to the total area of the stomach mucosa)[26]. The UI is determined using the following formula. UI = (Area of ulceration in $mm^2$/Area of stomach mucosa in $mm^2$) × 100. The UI is “0” when there is no ulcer. The person carrying out the analysis was not aware of the treatment the animal had received.

### Biochemical Analysis

Ten percent homogenates of the stomach were prepared using ice cold 0.05% Tween-20 in phosphate buffered saline (PBST); 1% homogenate was prepared when required by diluting 10% homogenate with PBST. The protein concentration in the homogenate was determined by the Lowry assay[28]. The lipid peroxidation, a marker for free radical–induced tissue injury[27] is estimated as TBARS by the method of Nichans and Samuelson[29]. The total SOD activity, Mn-SOD activity, and Cu-Zn SOD activity in the homogenate were measured using the xanthine/xanthine oxidase/cytochrome C method[30,31]. The CAT activity was assayed by the method of Sinha[32]. The GPX was estimated by the method of Rotruck et al.[33] with modifications. Reduced GSH was determined according to the method of Ellman[34] with modification.

### Estimation of GPX

The principle is to allow a known amount of enzyme preparation to react with $H_2O_2$ in the presence of reduced GSH for a specified time period. The remaining reduced GSH content was measured by the method of Ellman[34]. A mixture of 0.5 ml of 0.4 *M* $NaH_2PO_4$ (pH 7.0), 0.2 ml of 0.8 m*M* solution of ethylenediaminetetrasodium salt, 0.2 ml of sodium azide, 0.2 ml of GSH, 0.2 ml of $H_2O_2$, and 0.2 ml of distilled water and 0.5 ml of 10% tissue homogenate was incubated at 37ºC for 10 min along with a control containing all reagents except the homogenate. At the end of 10 min, the reaction was arrested by the addition of 1ml of 10% trichloroacetic acid. 0.5 ml of 10% tissue homogenate was added to the control alone after addition of 10% trichloroacetic acid. The tubes were centrifuged and the supernatant was assayed for reduced GSH. To 2 ml of supernatant, 8 ml of 0.3 *M* phosphate buffer and 1 ml of Ellman’ reagent prepared by dissolving 19.8 mg of 5,5-dithio(bis)nitrobenzoic acid (DTNB) in 100 ml of 1% sodium citrate solution (pH 8.0) are added. The yellow color developed is read at 412 nm immediately. A calibration curve based on five standards between 20 and 100 nmol/ml was used. The GPX activity was expressed as nmol of reduced GSH consumed/min/mg of protein.

### Estimation of GSH

It is based on the development of a yellow color due to reaction of DTNB with compounds containing sulfhydryl groups. One milliliter of 10% tissue homogenate was mixed with 1 ml of 5% trichloroacetic acid and then 1 ml of distilled water added. The precipitation was removed by centrifugation at 3000 rpm for 10 min. The supernatant was used for GSH assay. To 0.5 ml of the supernatant, 1 ml of 0.2 M of phosphate buffer (pH 8.0) and 2 ml of 0.6 mM DTNBsolution in phosphate buffer were added. The yellow color developed was read at 412 nm immediately. A calibration curve based on five standards between 20 and 100 nmol/ml of distilled water concentrations was used. The reduced GSH was expressed as nmol/mg of protein.

### Statistical Analysis

One-way analysis of variance (ANOVA) was used to compare the differences between the groups after taking logarithmic values. Further, least significant difference test was applied to find out which of the groups differed; $p$ values of <0.05 were considered as statistically significant.

## RESULTS

- Effects on gastric mucosa (Table 1) — Lesions were absent in control (Group 1) and taurine-treated (Group 2) rats (UI "0"). Administration of ibuprofen (Group 3) resulted in significant gastric mucosal lesions over the control group (Group 1) in the glandular portion of stomach (UI 2.67 ± 0.47 vs. 0.0 ± 0.0) at $p < 0.001$ level. In taurine cum ibuprofen–treated (Group 4) rats, there was a significant reduction in gastric mucosal lesions compared with ibuprofen-treated rats (Group 3) (UI 0.42 ± 0.30 vs. 2.67 ± 0.47) at $p < 0.001$ level.

**TABLE 1**
**Effects of Saline or Taurine in Normal or Ibuprofen-Treated Rats on UI, TBARS, Total SOD, Mn-SOD, Cu-Zn SOD, GPX, CAT, and GSH**

| | Group 1 | Group 2 | Group 3 | Group 4 |
|---|---|---|---|---|
| UI | 0.0±0.0 | 0.0±0.0 | 2.67±0.47$^{b*}$ | 0.42±0.30$^{a*}$ |
| TBARS | 30.34±1.65 | 32.93±6.30 | 31.69±5.98 | 22.14±4.39$^{a*}$ |
| Total SOD | 8.56±0.32 | 9.16±0.33$^{c*}$ | 9.84±0.27$^{b*}$ | 10.16±0.43$^{a‡}$ |
| Mn SOD | 8.31±0.44 | 8.87±0.22$^{c†}$ | 9.37±0.32$^{b*}$ | 9.81±0.63$^{a‡}$ |
| Cu-Zn SOD | 0.28±0.16 | 0.39±0.22 | 0.47±0.20 | 0.34±0.30 |
| GPX | 8.66±1.93 | 8.77±3.08 | 11.74±1.25$^{b*}$ | 14.57±0.89$^{a†}$ |
| CAT | 46.30±5.22 | 35.09±5.05$^{c*}$ | 45.24±3.21 | 56.70±7.26$^{a*}$ |
| GSH | 29.27±2.35 | 28.51±2.50 | 31.74±2.04$^{b‡}$ | 42.28±3.38$^{a*}$ |

[a]Ibuprofen treated vs. taurine cum Ibuprofen treated; [b]control vs. ibuprofen treated; [c]control vs. taurine treated.

*$p < 0.001$, †$p < 0.01$; ‡$p < 0.05$.

- Effects on lipid peroxidation (Table 1) — The lipid peroxidation status is estimated as the level of TBARS, which is expressed in nmol malondialdehyde (MDA)/g of wet tissue. Neither taurine nor ibuprofen treatment produced any significant difference in lipid peroxidation over control rats. However, there was a significant reduction in TBARS level in taurine cum ibuprofen–treated rats (Group 4) compared with the ibuprofen-treated rats (Group 3) (22.14 ± 4.39 vs. 31.69 ± 5.98 nmol MDA/g of wet tissue) at $p < 0.001$ level.
- Effects on total SOD activity (Table 1) — The total SOD activity is expressed in U/mg of protein. Administration of taurine (Group II) produced a significant increase in total SOD activity over the control rats (Group 1) (9.16 ± 0.33 vs. 8.56 ± 0.32 U/mg of protein) at $p < 0.001$ level. Ibuprofen treatment (Group 3) also induced a significant increase in total SOD activity over the control rats (Group 1) (9.84 ± 0.27 vs. 8.56 ± 0.32 U/mg of protein) at $p < 0.001$ level. Taurine (Group 4) increased the total SOD activity in ibuprofen-treated rats (Group 3) (10.16 ± 0.43 vs. 9.84 ± 0.27 U/mg of protein) at $p < 0.05$ level.

- Effects on Mn-SOD activity (Table 1) — The Mn-SOD activity is expressed in U/mg of protein. Taurine (Group 2) produced a significant increase in Mn-SOD activity over the control rats (Group 1) (8.87 ± 0.22 vs. 8.31 ± 0.44 U/mg of protein) at $p < 0.01$ level. Administration of ibuprofen (Group 3) produced a significant increase in Mn-SOD activity over the control rats (Group 1) (9.37 ± 0.32 vs. 8.31 ± 0.44 U/mg of protein) at $p < 0.001$ level. Mn-SOD activity in taurine cum ibuprofen–treated rats (Group 4) also showed a significant increase over ibuprofen-treated rats (Group 3) (9.81 ± 0.63 vs. 9.37 ± 0.32 U/mg of protein) at $p < 0.05$ level.
- Effects on Cu-Zn SOD activity (Table 1) — The Cu-Zn SOD activity is expressed in U/mg of protein. Treatment with saline or taurine or ibuprofen did not result in any significant change in the Cu-Zn SOD activity.
- Effects on CAT activity (Table 1) — The CAT activity is expressed as $H_2O_2$ consumed in µmol/min/mg of protein. Administration of taurine (Group 2) decreased CAT activity over the control rats (Group 1) (35.09 ± 5.05 vs. 46.30 ± 5.22 $H_2O_2$ consumed in µmol/min/mg of protein) at $p < 0.001$ level. Ibuprofen treatment did not have any effect on CAT activity. However, taurine cum ibuprofen-treated rats (Group 4) showed a significant increase in CAT activity over theibuprofen-treated rats (Group 3) (56.70 ± 7.26 vs. 45.24 ± 3.21 $H_2O_2$ consumed in µmol/min/mg of protein) at $p < 0.001$ level.
- Effects on GPX activity (Table 1) — The GPX activity is expressed as GSH consumed in nmol/min/mg of protein. Taurine administration (Group 2) did not produce any change in GPX activity over the control rats (Group 1). Treatment with ibuprofen (Group 3) increased the GPX activity over the control rats (Group 1) (11.74 ± 1.25 vs. 8.66 ± 1.93 GSH consumed in nmol/min/mg of protein) $p < 0.001$ level. Taurine cum ibuprofen–treated rats (Group 4) showed a significant increase in GPX activity over the ibuprofen-treated group (Group 3) (14.57 ± 0.89 vs. 11.74 ± 1.25 GSH consumed in nmol/min/mg of protein) at $p < 0.05$ level.
- Effects on reduced GSH level (Table 1) — The reduced GSH level is estimated in nmol/mg of protein. Treatment with ibuprofen (Group 3) resulted in a significant increase in reduced GSH level compared with the control rats (Group 1) (31.74 ± 2.04 vs. 29.27 ± 2.35 nmol/mg of protein) at $p < 0.05$ level. The reduced GSH level showed a significant increase in taurine cum ibuprofen–treated rats over the ibuprofen-treated group (42.28 ± 3.38 vs. 31.74 ± 2.4 nmol/mg of protein) at $p < 0.001$ level.

### DISCUSSION

In the present study, ibuprofen produced acute gastric mucosal lesions in the glandular portion of gastric mucosa. Oral administration of taurine prevented the gastric mucosal lesion produced by ibuprofen. Since taurine is an endogenous antioxidant, its protective role in ibuprofen-induced gastric mucosal lesion could be the outcome of its scavenging of free radicals. Son et al. have also found taurine to protect the gastric mucosa from indomethacin-induced gastric mucosal lesion in rats[14]. A number of studies on the protective role of other endogenous antioxidants in experimentally induced gastric mucosal lesions have been reported in the literature. Pihan et al. showed that SOD and CAT could significantly reduce the extent of gastric mucosal damage induced by aspirin[11]. Intravenous infusion of SOD or CAT is found to reduce mucosal damage induced by indomethacin in rats significantly[13]. Yoshikawa et al. have reported that treatment with SOD and SOD plus CAT significantly reduced the lesion area induced by indomethacin, but CAT alone showed no reduction of mucosal injury[7]. Similarly, the nutrient-derived antioxidants like vitamin E, vitamin C, and flavonoids also found to have the potential to reduce gastric mucosal injury in different experimental models.

Jaarin et al. have reported that palm vitamin E and tocopherol are effective in preventing the aspirin-induced gastric lesions in rats[18]. Palm vitamin E is also found to be effective in preventing ethanol-induced gastric injury in rats[16]. In another study by Sugimoto et al., vitamin E is found to prevent aspirin-induced gastric lesions in rats[17]. Acetic acid ulcers are found to be healed by vitamin C[20]. Pohle et al. have observed that vitamin C attenuated the gastric erosions induced by aspirin in human volunteers[19]. The flavonoids are a group of low molecular weight plant products based on the parent compound flavone (2-phenylchromone or 2-phenylbenzopyrone). Parmar and Parmar have reviewed the antiulcer potential of flavonoids in different experimental animal models of gastric ulcer[35]. Thus, the protective role of endogenous antioxidants in the treatment and/or prevention of gastropathies is another field of research that needs greater attention.

In many experimental studies, the level of lipid peroxidation is used as a marker for free radical–induced tissue damage[27]. In studies using indomethacin, aspirin, or diclofenac to induce gastric mucosal lesion, the lipid peroxidation is reported to be increased[7,14,36,37,38,39,40]. In our present study with ibuprofen, the lipid oxidation did not show any significant change. Oshima et al. have pointed out that lipid peroxidation may not be related to ulcer formation as observed in their ischemia-reperfusion experimental model to induce gastric mucosal lesion[41]. A similar observation is seen in our present study using ibuprofen. The significant decrease in lipid peroxidation observed in the taurine cum ibuprofen–treated group over the ibuprofen-treated group in the absence of any significant change in the lipid peroxidation between control and taurine treated or ibuprofen treated group needs to be studied in detail.

The cellular oxidative stress as a result of an imbalance between pro-oxidants and antioxidants status of the tissue is recently implicated in the pathogenesis of many disease states and in experimentally induced clinical conditions[10,42,43]. The oxidative stress may be the result of overproduction of ROS, inactivation of detoxification systems, consumption of antioxidants, and failure to adequately replenish antioxidants. The antioxidant enzymes of the tissue are particularly important for the primary endogenous defense against damaging actions of the ROS. The enzymatic defense against the ROS involves the cooperative action of three major intracellular antioxidant enzymes. The major defense against the toxicity of superoxide radicals is conferred by SODs. SOD catalyses the dismutation of superoxide radicals forming hydrogen peroxide. GPX and CAT are the unique enzymes scavenging hydroperoxides and therefore act in concert with SOD[44,45].

The expression of antioxidant enzymes is reported to be influenced by low levels of free radicals and the cytokines such as tumor necrosis factor (TNF)-α and interleukin (IL)-1β[46,47,48). In the present study, both taurine and ibuprofen administration resulted in increased activities of total SOD and Mn-SOD. Taurine as an antioxidant could be expected to produce a low level of free radicals that resulted in increased expression of Mn-SOD and total SOD. TNF-α is found to be increased following indomethacin-induced gastric mucosal damage[48]. Increase in the activities of total SOD and Mn-SOD following ibuprofen treatment could be the result of an expected increase in TNF-α for the NSAID. TNF-α is reported to upregulate Mn-SOD without affecting Cu-Zn SOD, CAT, or GPX in a human lung carcinoma cell line[49]. Ibuprofen treatment where an increase in the level of TNF-α is expected also did not produce any change in the activity of Cu-Zn SOD, but the activities of GPX and CAT showed an increase. The decrease in the CAT activity following taurine administration could be the result of an insufficient level of free radicals to stimulate its expression. Iqbal et al. have pointed out that in a population of patients with acute myocardial infarction, the erythrocyte showed an increased production of GSH and this response is said to be the result of mild oxidative stress[50]. The increase in the level of GSH of the stomach following ibuprofen treatment also could be the result of such a moderate oxidative stress.

In studies using indomethacin to induce gastric mucosal lesion, the total SOD, Mn-SOD, Cu-Zn SOD, CAT, GPX enzyme activities, and GSH have been reported to decrease[7,36,37,38]. However, in our study using ibuprofen, the total SOD, Mn-SOD, GPX, and CAT enzyme activities and GSH significantly increased and Cu-Zn SOD enzyme activity did not show any significant change. In another experimental model for gastric mucosal lesion in rats, use of 96% ethanol orally increased the SOD activity, but use of 0.6 *M* HCl, 0.2 *M* NaOH, or 25% NaCl produced a decrease in SOD activity[51]. Administration of 50% ethanol orally also was found to increase Mn-SOD, Cu-Zn SOD, and GPX enzyme activities in gastric mucosa[52]. In cultured rat gastric mucosal cells, aspirin did not affect cellular GSH or activities of the enzymes GPX or

CAT[39]. Thus, the values for antioxidant enzyme activities and the concentration of GSH following ROS-induced tissue damage reported in the literature varied from a decrease to an increase. Yet, in another study of rat ulcer model using ischemia-reperfusion technique, the authors have pointed out that endogenous free-radical scavengers may not correspond with tissue recovery[41]. Yamashita et al. have pointed out that a high dose of ROS causes damage to cardiac myocytes, whereas a low dose of such species acts as a signal transduction messenger in cells leading to an increase in antienzyme activity[53]. The contradictory results of ROS-induced tissue damage seen in various experimental models on the antioxidant status of the tissues could be the result of such effects. Thus, there appears to be no direct temporal relation between the tissue damage and its antioxidant status. However, the variation found in the temporal relation between the antioxidant status of the tissue and the tissue damage could also be the outcome of the different experimental designs used by the authors. Therefore, detailed studies are needed before any conclusion is drawn on the nature of the relation between the free radical–induced tissue damage and its antioxidant status.

In the present study, oral intake of the antioxidant taurine (250 mg/kg body weight) for three consecutive days prevented the acute gastric mucosal lesion following oral administration of ibuprofen in rats. The antioxidant status of the stomach in terms of total SOD, Mn-SOD, GPX, CAT, and GSH is also influenced by ibuprofen and taurine. The Cu-Zn SOD is not affected. There appears to be no temporal relation between the antioxidant status of the stomach and the tissue damage.
